# Effect of Stocking Density on Behavioural and Physiological Traits of Laying Hens

**DOI:** 10.3390/ani15040604

**Published:** 2025-02-19

**Authors:** Kamila Janicka, Kamil Drabik, Karolina Wengerska, Iwona Rozempolska-Rucińska

**Affiliations:** Institute of Biological Basis of Animal Production, University of Life Sciences in Lublin, 20-950 Lublin, Poland; kamil.drabik@up.lublin.pl (K.D.); karolina.wengerska@up.lublin.pl (K.W.); iwona.rucinska@up.lublin.pl (I.R.-R.)

**Keywords:** laying hens, stocking density, behaviour, stress hormones

## Abstract

This study analysed stocking density as a major stress factor for laying hens and assessed its impact on selected behavioural and physiological traits of the birds. The study population included 142 birds of the Green-legged Partridge breed (12 males and 132 females). The birds were randomly assigned to three experimental groups of standard, low, and high stocking density, and a modified open-field test was conducted to assess their behavioural traits. The following blood parameters were analysed: corticosterone, cortisol, and testosterone. The behavioural test results demonstrated an impact of stocking density on laying hens’ behaviour. The birds kept at a low density exhibited the greatest diversity in behaviour as well as the most frequent and longest locomotion. There were no differences between the groups in terms of stress hormone results. All groups showed a significant decrease in testosterone levels compared to the control. This may suggest that, despite differences in stocking, the structure was established quickly, resulting in decreased competition and, consequently, a positive impact on the birds. When kept in lower-stocking-density groups with a stable structure, birds are less competitive; this stable structure improves their well-being to a greater extent than the stocking density.

## Simple Summary

The presence of stressors is common in hens. Stocking density seems to play a special role in poultry production. Changes in stocking density are reflected in behavioural and physiological traits. The present study aimed to investigate the effects of stocking density on the behavioural and physiological traits of laying hens. The study hypothesized that stocking density could affect the behaviour of hens, with the expectation that hens in the low-stocking-density group would be more active and interested in the test environment. In addition, higher stocking densities were expected to create a more stressful environment, which would be reflected in blood parameters. Birds kept at low densities showed the greatest variety in behaviour, as well as the most frequent and longest locomotion. There were no differences between groups in terms of stress hormone scores. All groups showed a significant decrease in testosterone levels compared to the control group. The changes observed in the birds’ behaviour indicate the possible effect of stocking density on their welfare. When hens are free to move around and explore their environment, they exhibit a wider range of behaviours in a new location.

## 1. Introduction

Rapid and unpredictable changes may have significant effects on survival and coping ability. In order to survive, animals need to gain information about the environment [1,2]. The concept of animal welfare developed by Ohl and van der Staay [3] states: “An individual is in a positive welfare state when it is able to actively adapt to its living conditions and to reach the state that it perceives as a positive” [4]. Adaptation to change may be a variable trait and is likely to be influenced by conditions during an individual’s development [1,2]. Adaptation is the ability of any living organism to survive and reproduce successfully under the existing environment [5]. In a complex environment, birds must adapt to changing circumstances (both negative and positive) [6,7,8]. There is a growing research interest aimed at assessing personality, as it is important to understand how and why animals differ in their reactions despite facing similar life conditions [9]. Some birds have little or no response to stressors that evoke a relatively large response in other birds [6]. If the changes are potentially harmful, the animals adapt to them by generating appropriate reactions [6,7,10]. Diverse coping styles may, to a certain extent, be attributed to differences in stress hormone profiles. Proactive individuals have a more pronounced sympathetic stress activation (flight or fight), whereas reactive individuals often respond to a stressful situation with a higher level of parasympathetic stress activation (withdrawal response) [11]. However, the expression of adaptive behaviour depends on a certain optimal stress level [7]. Strong and/or prolonged stress weakens the activity of all physiological structures responsible for the adaptation process [12].

The presence of stressors is common for hens, and birds are exposed to stressors such as stocking density, temperature, food limitations, transport, and pollution [13]. The first two are among the most important factors in the poultry industry [13,14,15]. However, stocking density seems to play a special role [13,16,17]. Animal welfare largely depends on the possibility of the animal exhibiting natural behaviour [18], which, in hens, is associated with sufficient living space [16,17]. Keeping too many birds per square meter (m^2^) can seriously impact their welfare [19,20,21]. Changes in stocking density are reflected in behavioural and physiological traits [13,22]. A restricted breeding environment can significantly limit or even eliminate the manifestation of natural behaviour in hens [22]. When the space is overcrowded, it can lead to discomfort and anxiety, which can result in changes in behaviour [21]. In such conditions, the laying hens’ activity decreases [15], their comfort behaviour is limited [23], and the use of cage elements is reduced, which can lead to lower consumption of feed and water and result in injuries and diseases. Furthermore, an increase in aggressive behaviour and pecking out feathers is observed [23]. In addition to changes in behaviour, there may be signs of physiological stress [15], such as alterations in leukocyte [12] and corticosterone levels [13,22]. In birds, the main glucocorticoid is corticosterone. This hormone can affect multiple regulatory and behavioural changes [23,24] that are thought to help birds to adjust to stressful situations [7]. Therefore, stocking density is crucial in poultry management as it impacts welfare, productivity, and economic viability [16,17,25]. Continuous improvement is essential in poultry production, so it is important to implement standards that optimise the welfare of poultry. The rearing environment can present poultry with a number of challenges that can lead to frustration. Understanding these challenges makes it possible to adapt the environment to the needs of the animals. This allows birds to control the environment to a certain point [26].

The continued study of stress in relation to stocking density is important. In research and industry, the use of the Green-legged Partridge is becoming more common. Due to increasing pressure from high-producing commodity hybrids, bird breeds with lower productivity have lost ground. Due to the need to protect their often-unique characteristics, some breeds were included in the Conservation of Genetic Resources (CYT) Programme. One of the breeds protected is the Green-legged Partridge [27]. The Green-legged Partridge is one of the oldest breeds of hen kept in Europe [28]. Due to the need to maintain generational continuity in the primogenitor of this breed, intensive breeding work is not applied, but rather, work is undertaken to preserve the genotype [27]. This makes the birds of this breed a valuable research material both for studies of the behaviour of hens and their physiology [29] and of the quality of the raw materials obtained [28]. Although they are not a typical commercial hybrid, their popularity is increasing due to their high adaptability to extensive rearing and also the reduced cholesterol content of their egg yolks [28]. Furthermore, due to their maintenance under conservative breeding conditions, they have become an excellent model for behavioural studies as a reference for breeds and lines undergoing intensive breeding work [29,30]. In addition, due to the qualities of the eggs obtained from them, the popularity of this breed is steadily increasing, and selection lines to be used as laying hens have already been produced.

This study aimed to investigate the effect of stocking density on certain behavioural and physiological traits in laying hens. The study assumed that stocking density could influence the behaviour of hens during the test, with hens in the low-stocking-density group expected to be more active and interested in the test environment. Additionally, higher stocking density was expected to create a more stressful environment, which would be reflected in blood parameters.

## 2. Materials and Methods

### 2.1. Animals and Housing

The study was approved by the Local Ethics Committee for Animal Experiments acting at the University of Life Sciences in Lublin, Poland (no. 69/2017).

The study population included 142 birds (12 males and 132 females) of the Green-legged Partridge breed aged 45 weeks. The birds were randomly selected from four separate flocks kept in the same building, in identical zoohygienic conditions, and they were combined to make experimental flocks on the first day of the experiment. The birds were divided into three experimental groups:

- standard—kept at the standard stocking density for reproductive flocks (6 birds/m^2^),
- low—kept at a low stocking density (3 birds/m^2^)
- high—kept at a high stocking density (9 birds/m^2^)

For experimental purposes, the groups of birds were kept for 21 days in pens with dimensions of 2 m^2^, 1.5 m^2^, and 4 m^2^, respectively (4 replications in each; the total number of experimental pens *n* = 12), equipped with automatic drinking and feeding lines. Each box was equipped with nest boxes, with each box containing 4 individual nests. The material for bedding the nests was hay. The area of the nest box was not included in the usable area of the box. There were 12 birds in each pen (1 male and 11 females; this sex ratio is typical of breeding herds). To avoid inflicting potential stress on the laying hens related to the absence of a male in the group, it was decided to leave the roosters in the experimental pens. The roosters were not assessed for physiological and behavioural parameters. The birds had continuous access to water and a fodder mix suited to their age. The birds were kept in a litter system (straw litter) with access to four individual nests per pen. The lighting scheme typical of laying hens (16 h light: 8 h darkness) was followed throughout the experiment, and the temperature was maintained at 20 °C. LED lighting was used (4000 K, intensity 80 lx).

### 2.2. Blood Samples

On the first day of the experiment (measurement labelled as control) and after 21 days (measurements for various stocking densities labelled as low, standard, high) of keeping the birds in experimental conditions, the corticosterone, cortisol, and testosterone levels were measured by drawing blood from the wing vein to vacuum tubes with an anticoagualant K3-EDTA (MEDLAB-PRODUCTS, Ldt., Raszyn, Poland) from 12 randomly selected laying hens in each group (total number *n* = 36). After sampling, the tubes were placed on a hematologic stirrer and centrifuged on a laboratory centrifuge for 10 min at 3000 rpm. Next, serum was collected and taken for further analysis. The levels of testosterone [pg/mL], cortisol [pg/mL], and corticosterone [pg/mL] were measured with commercial Enzyme-Linked Immunosorbent Assay kits (ELISA kits, Qayee Biotechnology Co., Ltd., Shanghai, China). The calibration curve preparation and the measurement procedure were in line with the test kit producer’s guidance. The analysis was performed with 10 µL of serum, and each sample was analysed in triplicate, with the result being the mean of the three measurements. The results were read with the Biotek Synergy H1 microplate reader ((BioTek Instruments, Inc., Winooski, VT, USA) at the wavelength of λ = 450 nm.

### 2.3. Behavioural Test

**Figure 1:**
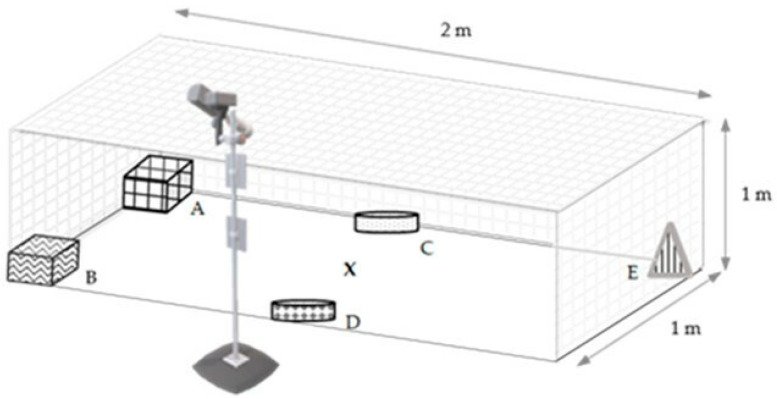
Experimental pen with elements and camera; A—nest imitation; B—sandpit; C—commercial fodder; D—enriched fodder; E—water trough; X—localisation of laying hens’ ‘start position’.

Individual behavioural tests, based on that described by Kozak et al. [31], were conducted on 12 randomly selected hens for each experimental group (standard *n* = 12; low *n* = 12; high *n* = 12; total number of hens tested during individual behavioural test *n* = 36). The tests were conducted on two consecutive days (Days 21 and 22) between 8.00 a.m. and 4.00 p.m. The impact of bird immobilisation during blood drawing on a behaviour change was eliminated by the test being conducted only on birds from which samples had not been taken. Moreover, the birds’ access to fodder was not limited on the test day to prevent the impact of this factor. The test pen was situated in the same building as the experimental pens. However, it was placed in an adjoining room with identical lighting and heating conditions. This prevented the birds from making eye or sound contact with other individuals during the test. Elements unknown to birds were placed along the longer walls of the test pen. They included a container that imitated a nest (A), a sandpit (B), a container with commercial fodder (fodder adapted to the needs of laying hens, used in breeding) (C), a container with enriched fodder (commercial fodder supplemented with herbs) (D), and a drinking trough (E) (Figure 1).

An experimenter known to the hens transferred the bird being tested directly from the experimental pen to the test one (at its central point X). After the pen was closed, the experimenter switched on the camera, exhibited a plate with the number of birds being tested (No. 1–36), and subsequently left the room. The behaviour was recorded (660 s) with a wide-angle camera (Xblitz MOVE 4K Plus, KGK Trend, Ltd., Kraków, Poland) placed behind the test pen. Due to the presence of some factors (the view of the experimenter leaving the room, the sound of footsteps, and the closing door), which could have affected the bird’s reactions, the initial section of the recording was deleted. An analysis of the recording lasting 600 s was performed in the BORIS (version 7.10.2., 2021) software, following Table 1. The observer was blind to the density group of the birds observed in a given recording.

### 2.4. Statistics

The normality of the distribution was checked using the Shapiro–Wilk test (PROC UNIVARIATE procedure). Since the obtained data were not normally distributed, a non-parametric analysis was used as the F-approximation of the Friedman test and the associated rank-sum multiple comparison test [32]. The effects of the experimental groups (standard density, low density, high density) were analysed with PROC GLIMMIX (SAS software Statistical Analysis System, 9.4).

## 3. Results

### 3.1. The Impact of Stocking Density on Hen Behavioural Traits

The mean time [s] and frequency [freq.] of the behaviours under study that were exhibited in each group are shown in Table 2. Significant inter-group differences were shown for latency 2, locomotion_t, locomotion_f, water_t, water_f, and o2l (Table 1). The medium time and frequency of the other attributes (fodder 1_t, fodder 1_f, fodder 2_t fodder 2_f, sandpit_t, sandpit_f, water_f, nest_t, and nest_f) accounted only for a portion part of overall activity during the test. Since the inter-group differences (Table S1) for these attributes were not significant, they are not shown in Table 3.

Compared with those in the standard-stocking-density group (standard), birds in the low-density group (low) needed much more time before they started to move towards any element in the test pen (*p* = 0.027) (Table 3). The locomotion time was significantly shorter for the birds in the standard- (*p* = 0.039) and high-stocking-density (*p* = 0.015) groups than that observed in the birds in the low-stocking-density group. Moreover, birds in the latter group initiated any locomotor activity significantly more frequently (*p* = 0.044) than those in the high-density group. Compared to the birds in the standard-density group, the birds in the low-density group drank water for a longer time, stayed close to the drinking trough more often (*p* = 0.010), and approached it more frequently (*p* = 0.036). The ratio of time spent at enriching elements to the locomotion time for birds in the high-density group was higher than that for the birds in the standard- (*p* = 0.030) and low-stocking-density (*p* = 0.021) groups.

### 3.2. The Impact of Stocking Density on Hen Physiological Traits

**Figure 2:**
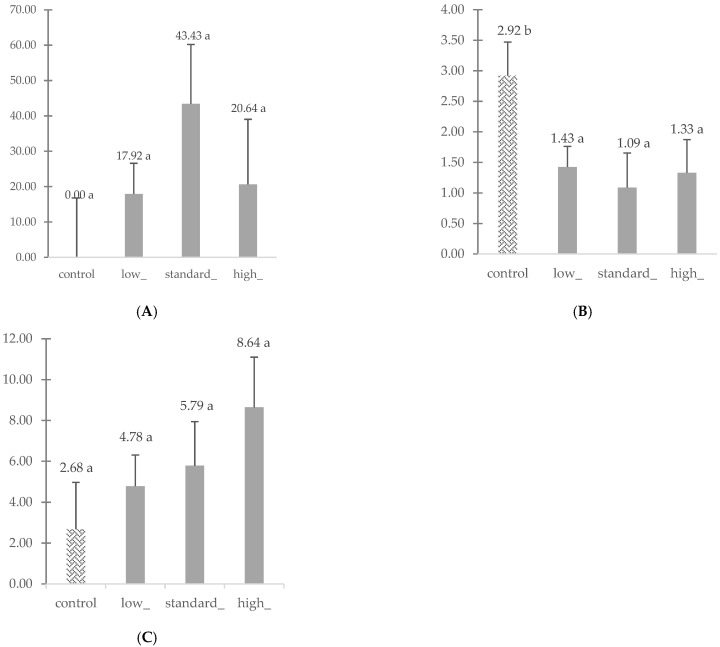
(**A**) Mean concentration (LSM + SE) of corticosterone (pg/mL), (**B**) testosterone (pg/mL), and (**C**) cortisol (pg/mL) in the hens’ blood before the experiment (control) and after three weeks in various stocking density groups (low, standard, high). Means marked with different letters (a, b) differ significantly at *p* < 0.05.

The mean levels of corticosterone [pq/mL], testosterone [pq/mL], and cortisol [pq/mL] are shown in Figure 2. Significant differences were observed only for testosterone. The mean testosterone level was significantly lower in birds in all the groups (*p* = 0.022, *p* = 0.023, *p* = 0.046) compared with the control. However, no significant differences were observed between birds in the low-, high-, and standard-stocking-density groups. No significant differences were demonstrated for cortisol or corticosterone, either between different density groups (low, standard, high) or compared to the pre-experiment levels (control).

## 4. Discussion

The current study revealed that behaviour may indicate the effect of stocking density on bird comfort. Hens are eager to explore their environment and display a wider range of behaviours when they have access to more space. However, in order to evaluate the welfare of laying hens, it is necessary to consider not only the measurement of behavioural indicators but also physiological and physical indicators. Approaches to animal welfare management are moving away from the concept of adapting the animal to its environment to the concept of adapting the environment to the animal through the planning of appropriate conditions. This is partly related to the fact that external factors are much easier to control and adjust than internal factors [4]. Continuous improvement toward the implementation of the highest standards is a key element in the optimization of animal welfare. Many factors that contribute to the welfare of laying hens affect the success of the poultry industry [16]. According to Broom’s concept of animal welfare [29], when the coping capacity of the individual is exceeded, the result is poor welfare. However, this is a major oversimplification; the modern definition of welfare considers much more than just the ability to cope [4].

Corticosterone secretion increases when a bird perceives a stressor. This major glucocorticoid hormone in birds promotes changes in behaviour and metabolism that are thought to help birds to adjust to stressful situations [4,5,6,7]. Assessing an individual’s perception of its emotional state requires a holistic view and ideally combines physiological and behavioural measures. Physiological indicators of arousal or stress management, such as glucocorticoid levels, can be valuable indicators of welfare [4]. The physiological response to acute or chronic stress is examined routinely by measuring the level of corticosterone [22] or cortisol as an indicator of hypothalamic–pituitary–adrenal (HPA) axis activity [33], although classically, in bird studies, the parameter recorded is corticosterone. However, it is also possible to analyse cortisol levels [33,34]. Despite the fact that stocking density is considered to be one of the most stressful factors in animal production [13,14,17], this study did not reveal significant differences in cortisol or corticosterone levels. It might seem that different densities did not influence the level of stress at a given point in time, but this is not necessarily the case. It is also noteworthy that a lack of change in the levels of the examined hormones does not necessarily indicate a stress-free environment. Cockrem [7] points out that responses to corticosterone are usually presented in as average responses of groups of birds. However, there is significant variation between individuals in the pattern of their responses to corticosterone, and individual responses to corticosterone may differ significantly from the average response of a group of birds. Our study focused on the mean levels for the group and disregarded hens’ individual responses. The large standard errors for the groups in the study indicate that the values for each bird varied widely. In fact, the results might have been different if every hen had been assessed separately. Admittedly, in our study, we focused on the results for the group and did not treat the birds individually, but it is likely that the behaviour of the birds might have influenced the findings [35], so it would be beneficial to consider individual assessment for future research. If we are to strive to improve well-being, then we need to start paying attention to the individual and not just the whole group.

When it comes to assessing behavioural indicators, the expression of normal behaviour does not necessarily equate to good welfare [4]. Therefore, when assessing welfare, both physiological and behavioural measurements should be taken into account, as they will better represent the actual condition of the animal. Being social animals, laying hens live in a complex and demanding environment [7]. Because of this, hens exhibit various ways of coping with environmental changes [7,9], which are dynamic processes [4] that can be attributed to a certain extent to differences in stress hormone profiles [11,23]. Kang et al. [22] indicated that variable stocking density, the establishment of a hierarchy, and competition lead to increased corticosterone secretion. It has been suggested that testosterone is the most important hormone for preparing individuals for intense social competition. Dominant individuals, who tend to be more aggressive, are most likely to be successful in competition by better focusing attention on the target (e.g., food source). The highest levels of testosterone are found in such birds [36]. The lack of variation in the corticosterone response found here may suggest that the hens were not under stressful conditions that would require them to compete for resources. This is supported by the significant decrease in testosterone levels across all groups compared to the initial measurements taken on the first day of the experiment. Laying hens kept in small groups, as in this experiment, exhibit social behaviours similar to those of their wild ancestors, and the social organisation is stable. In stable groups, the social structure tends to stabilise to avoid the costs and risks associated with increased and constant fighting. Enabling the development and maintenance of stable connections can create a positive social environment and improve the ability to cope with new stressors [37]. It seems that the examined hens were likely to accept the leading role of the rooster and the structure of the group, which is why testosterone levels were lower than in the control group [36]. Therefore, it is worth including this aspect in future research. The hormones measured are an essential indicator to be taken into account in research on the welfare of laying hens. However, it should be emphasized that assessing welfare status requires multiple measurements over time, as this will reflect the dynamics of an individual’s interaction with the environment. The number of measurements used in our study is a limitation, as it only allowed us to determine the momentary state of the animal. Nevertheless, the results are valuable as they provide an indication of urgent action being taken to improve the condition.

Behaviour is an essential indicator of an animal’s welfare status, and it allows the identification of changes [4]. Many authors [10,12,13,15,23] have indicated that stressors can modify birds’ behaviour as well as changing their physiological traits. The behavioural test results in hens revealed certain differences in behaviours such as locomotion or the use of environmental elements. The effect of the stocking density is rather apparent in the former. The birds in pens with both high and standard stocking densities moved less than those kept at a lower density, which also moved more frequently. Other authors have observed similar effects [15,16,38]. Van der Eijk [38] found that living space impacts the level of activity, feeding, and comfort of birds, which is a consequence of access to free space, a better quality of litter, and the ability to walk. The presence of proper litter is one of the most important factors and integral elements in ensuring a better environment for fast and healthy growth [39]. This is of particular importance in young, developing birds [40]. One could argue that when birds live in a spacious area with low population density, they have more freedom and curiosity to explore their surroundings. Conversely, when birds are crowded together in a high-density environment, their movement is restricted, so meeting their basic needs becomes more challenging. This is why they can tend to spend less time exploring unfamiliar environments.

It seems worthwhile to include an analysis of the time spent on using elements of the environment to assess the impact of stocking density, as this can be associated with birds’ general motivation. This study demonstrated an increased frequency of instances and time spent on water consumption by birds kept at a lower density than those in the standard-density group. Moreover, the ratio of the time spent at enriching elements to the locomotion time was lower in birds kept in the lowest- and standard-density pens compared to those in the high-density pen. These observations suggest that high density limits the variety of behaviours and exploration of hens. Unlike birds in the highest-density group, those in the other two groups (the standard- and the low-density groups) exhibited a greater need for movement and explored their environment more thoroughly. This may show that hens felt more confident and comfortable in these conditions. These observations suggest that high density limits the variety of behaviours and exploration of hens. Birds of various breeds can show different behavioural and physiological responses to stimuli. The fact that the hens were not seen to be interested in the elements in the test pen can indicate that the breed under study has different preferences [41]. Interestingly, the standard-density group, rather than the low-density group, needed much less time to approach any element, so, in theory, they were more willing to explore. According to the authors, such behaviour can be attributed to increased motivation caused by competing for resources, which would not necessarily indicate a positive affective state.

## 5. Conclusions

Our study led us to the conclusion that the behaviour observed in the study could indicate the effect of stocking density on the birds’ comfort. When hens are free to move and explore their environment, they exhibit a wider range of behaviours in a new location. However, the increased activity may be due to their desire to return to an environment that satisfies their needs. Although the blood results obtained provided little support for behavioural indicators, we want to emphasise that we need to take a comprehensive approach when assessing welfare. In order to demonstrate a permanent deterioration in animal welfare, numerous measurements of behavioural, physiological, and physical indicators over a long period of time must be taken into account. It should be noted that the responses of individual birds may differ significantly from the average response of a group of birds. Therefore, the effectiveness of welfare interventions may be enhanced by knowledge of individual behavioural and physiological response norms.

## Figures and Tables

**Table 1:** Behaviours assessed during the modified open field test.

Trait	Description
latency 1 [s]	Latency time to start locomotion
latency 2 [s]	Latency time to approach the random element
locomotion_t [s]	Total time spent on locomotion (laying hen takes a minimum of two steps)
locomotion_f [freq.]	Total number of repetitions of the locomotion episodes
fodder 1_t [s]	Total time spent on feed intake/staying in direct proximity of the container filled with commercial fodder
fodder 1_f [freq.]	Total number of repetitions of the feed intake/staying in direct proximity to the container filled with commercial fodder
fodder 2_t [s]	Total time spent on feed intake/staying in direct proximity to the container filled with enriched fodder
fodder 2_f [freq.]	Total number of repetitions of feed intake/staying in direct proximity to the container filled with enriched fodder
water_t [s]	Total time spent on water intake/staying in direct proximity to the watering trough
water_f [freq.]	Total number of repetitions of water intake/staying in direct proximity to the watering trough
sandpit_t [s]	Total time spent dust bathing/staying in direct proximity to the sandpit
sandpit_f [freq.]	Total number of repetitions of dust bathing/staying in direct proximity to the sandpit
nest_t [s]	Total time spent staying in the nest imitation/staying in direct proximity to the nest imitation
nest_f [freq.]	Total number of repetitions of staying in the nest imitation/staying in direct proximity to the nest imitation
o2l	The ratio of the average time spent at the elements in the test pen to the average time of locomotion

**Table 2:** Mean time in seconds and frequency [freq.] of behaviours evaluated during the behavioural test.

Trait	Density	Estimate	SE	Lower	Upper
latency 1	standard	221.94	61.83	96.14	347.74
	high	264.50	61.83	138.70	390.30
	low	156.90	61.83	31.10	282.70
latency 2	standard	349.40	59.12	229.12	469.68
	high	277.15	59.12	156.87	397.43
	low	155.23	59.12	34.95	275.51
locomotion_t	standard	33.50	15.67	1.61	65.38
	high	24.50	15.67	−7.38	56.39
	low	81.14	15.67	49.25	113.02
locomotion_f	standard	8.25	2.61	2.93	13.57
	high	7.08	2.61	1.77	12.40
	low	14.83	2.61	9.52	20.15
fodder1_t	standard	11.09	6.29	−1.71	23.90
	high	12.63	6.29	−0.18	25.43
	low	10.29	6.29	−2.51	23.09
fodder1_f	standard	1.75	1.39	−1.08	4.58
	high	2.92	1.39	0.09	5.75
	low	2.50	1.39	−0.33	5.33
fodder2_t	standard	10.54	5.59	−0.82	21.91
	high	9.08	5.59	−2.29	20.45
	low	9.17	5.59	−2.20	20.53
fodder2_f	standard	0.92	0.60	−0.31	2.14
	high	1.00	0.60	−0.23	2.23
	low	1.67	0.60	0.44	2.89
water_t	standard	13.04	6.05	0.73	25.34
	high	20.38	6.05	8.07	32.68
	low	36.58	6.05	24.27	48.88
water_f	standard	6.92	2.84	1.14	12.69
	high	10.83	2.84	5.06	16.61
	low	15.67	2.84	9.89	21.44
sandpit_t	standard	3.40	19.71	−36.71	43.51
	high	40.33	19.71	0.22	80.44
	low	4.62	19.71	−35.49	44.73
sandpit_f	standard	0.17	0.55	−0.95	1.28
	high	1.08	0.55	−0.03	2.20
	low	1.08	0.55	−0.03	2.20
nest_t	standard	1.52	2.91	−4.40	7.44
	high	4.04	2.91	−1.88	9.96
	low	5.01	2.91	−0.91	10.93
nest_f	standard	0.08	0.45	−0.84	1.00
	high	0.33	0.45	−0.59	1.25
	low	0.83	0.45	−0.09	1.75
o2l	standard	1.27	1.05	−0.90	3.43
	high	4.69	1.05	2.52	6.85
	low	1.20	0.95	−0.76	3.15

SE—standard error; lower—lower confidence interval; upper—upper confidence interval

**Table 3:** Estimators of differences in exhibition of selected behaviours by hens depending on the stocking density.

Trait	Density	_Density	Estimate	SE	*p*	Lower	Upper
latency 2	standard	low	194.17	83.61	0.027	24.07	364.26
locomotion_t	standard	low	−47.64	22.16	0.039	−92.73	−2.55
locomotion_t	high	low	−56.63	22.16	0.015	−101.72	−11.54
locomotion_f	high	low	−7.75	3.69	0.044	−15.27	−0.23
water_t	standard	low	−23.54	8.55	0.010	−40.94	−6.14
water_f	standard	low	−8.75	4.01	0.036	−16.92	−0.58
o2l	standard	high	−3.42	1.49	0.030	−6.48	−0.36
o2l	high	low	3.49	1.42	0.021	0.57	6.40

SE—standard error; *p*—probability value; lower—lower confidence interval; upper—upper confidence interval.

## Data Availability

The original contributions presented in this study are included in the article/Supplementary Materials. Further inquiries can be directed to the corresponding author.

## Supplementary Materials

The following supporting information can be downloaded at: https://www.mdpi.com/article/10.3390/ani15040604/s1, Table S1: Estimators of differences in exhibiting selected behaviours by hens depending on the stocking density.
