# Supplementary material for: Effect of Stocking Density on Behavioural and Physiological Traits of Laying Hens
Source: Animals (Basel). 2025 Feb 19;15(4):604. doi: 10.3390/ani15040604 (PMC11852137; doi:10.3390/ani15040604)
Supplement: Supplementary file 1 [file animals-15-00604-s001.zip › animals-3310859-supplementary.pdf]

Supplement material Table S1. Estimators of differences in exhibiting selected behaviours by hens depending on the stocking density.

Trait	Density	_Density	Estimate	SE	*p*	Lower	Upper
latency 1	standard	high	-42.56	87.44	0.630	-220.47	135.34
latency 1	standard	low	65.04	87.44	0.462	-112.86	242.94
latency 1	high	low	107.60	87.44	0.227	-70.30	285.51
latency 2	standard	high	72.25	83.61	0.394	-97.85	242.35
latency 2	standard	low	194.17	83.61	0.027	24.07	364.26
latency 2	high	low	121.92	83.61	0.154	-48.18	292.01
locomotion_t	standard	high	9.00	22.16	0.687	-36.10	54.09
locomotion_t	standard	low	-47.64	22.16	0.039	-92.73	-2.55
locomotion_t	high	low	-56.63	22.16	0.015	-101.72	-11.54
locomotion_f	standard	high	1.17	3.69	0.754	-6.35	8.68
locomotion_f	standard	low	-6.58	3.69	0.084	-14.10	0.93
locomotion_f	high	low	-7.75	3.69	0.044	-15.27	-0.23
fodder 1_t	standard	high	-1.53	8.90	0.864	-19.64	16.57
fodder 1_t	standard	low	0.80	8.90	0.929	-17.30	18.91
fodder 1_t	high	low	2.33	8.90	0.795	-15.77	20.44
fodder 1_f	standard	high	-1.17	1.97	0.557	-5.17	2.84
fodder 1_f	standard	low	-0.75	1.97	0.705	-4.75	3.25
fodder 1_f	high	low	0.42	1.97	0.834	-3.59	4.42
fodder 2_t	standard	high	1.46	7.90	0.854	-14.62	17.54
fodder 2_t	standard	low	1.38	7.90	0.863	-14.70	17.46
fodder 2_t	high	low	-0.08	7.90	0.992	-16.16	15.99
fodder 2_f	standard	high	-0.08	0.85	0.923	-1.82	1.65
fodder 2_f	standard	low	-0.75	0.85	0.385	-2.48	0.98
fodder 2_f	high	low	-0.67	0.85	0.439	-2.40	1.07
water_t	standard	high	-7.34	8.55	0.397	-24.74	10.06
water_t	standard	low	-23.54	8.55	0.010	-40.94	-6.14
water_t	high	low	-16.20	8.55	0.067	-33.60	1.20
water_f	standard	high	-3.92	4.01	0.336	-12.08	4.25
water_f	standard	low	-8.75	4.01	0.036	-16.92	-0.58
water_f	high	low	-4.83	4.01	0.237	-13.00	3.33
sand_t	standard	high	-36.94	27.88	0.194	-93.66	19.79
sand_t	standard	low	-1.23	27.88	0.965	-57.95	55.50
sand_t	high	low	35.71	27.88	0.209	-21.02	92.43
sand_f	standard	high	-0.92	0.78	0.246	-2.50	0.66
sand_f	standard	low	-0.92	0.78	0.246	-2.50	0.66
sand_f	high	low	0.00	0.78	1.000	-1.58	1.58
nest_t	standard	high	-2.52	4.11	0.544	-10.89	5.85
nest_t	standard	low	-3.49	4.11	0.402	-11.86	4.88
nest_t	high	low	-0.97	4.11	0.81	-9.34	7.40
nest_f	standard	high	-0.25	0.64	0.699	-1.55	1.05
nest_f	standard	low	-0.75	0.64	0.250	-2.05	0.55
nest_f	high	low	-0.50	0.64	0.441	-1.80	0.80
o2l	standard	high	-3.42	1.49	0.030	-6.48	-0.36
o2l	standard	low	0.07	1.42	0.963	-2.85	2.98
o2l	high	low	3.49	1.42	0.021	0.57	6.40

SE – standard error; *p* – probability value; Lower – lower confidence interval; Upper – upper confidence interval
